# Merotelic attachments and non-homologous end joining are the basis of chromosomal instability

**DOI:** 10.1186/1747-1028-5-13

**Published:** 2010-05-17

**Authors:** Astrid Alonso Guerrero, Carlos Martínez-A, Karel HM van Wely

**Affiliations:** 1 Department of Immunology and Oncology, Centro Nacional de Biotecnología/CSIC, Darwin 3, UAM Campus Cantoblanco, 28049 Madrid, Spain

## Abstract

Although the large majority of solid tumors show a combination of mitotic spindle defects and chromosomal instability, little is known about the mechanisms that govern the initial steps in tumorigenesis. The recent report of spindle-induced DNA damage provides evidence for a single mechanism responsible for the most prominent genetic defects in chromosomal instability. Spindle-induced DNA damage is brought about by uncorrected merotelic attachments, which cause kinetochore distortion, chromosome breakage at the centromere, and possible activation of DNA damage repair pathways. Although merotelic attachments are common early in mitosis, some escape detection by the kinetochore pathway. As a consequence, a proportion of merotelic attachments gives rise to chromosome breakage in normal cells and in carcinomas. An intrinsic chromosome segregation defect might thus form the basis of tumor initiation. We propose a hypothesis in which merotelic attachments and chromosome breakage establish a feedback loop that results in relaxation of the spindle checkpoint and suppression of anti-proliferative pathways, thereby promoting carcinogenesis.

## Introduction

**Figure 1:**
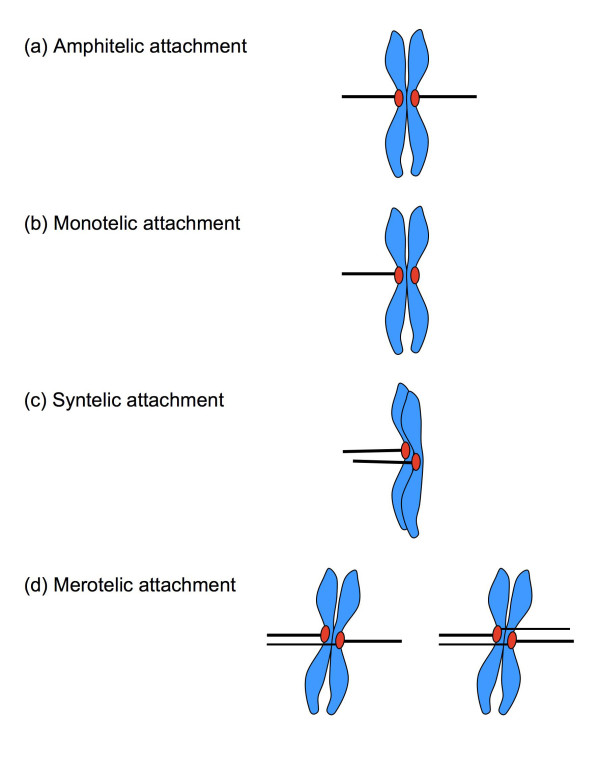
**Spindle attachment defects**. (a) In amphitelic attachment, the sister kinetochores are correctly connected to microtubules from opposite poles, resulting in a bioriented chromosome. (b) In a monotelic attachment, only one of the sister chromatids is connected to a spindle pole; the chromosome is mono-oriented. (c) In a syntelic attachment, both sister kinetochores are attached to a single spindle pole, and the chromosome is mono-oriented. (d) In a merotelic attachment, usually one or, rarely, both sister kinetochores are connected to both poles instead of one. Chromosomes are bioriented in merotelic attachments.

Mitosis comprises a brief period of intense activity in the cell cycle. The segregation of sister chromatids into daughter cells involves moving the largest molecules encountered in nature (the chromosomes) over distances greater than the size of most organelles. To ensure sufficiently rapid chromosome segregation, most eukaryotes connect each centromere to a bundle of parallel microtubules, termed the kinetochore fiber, along which an outward-pulling force moves sister chromatids towards the spindle poles [1]. Chromosome segregation must be completed quickly, since mitosis represses other cell functions [2-4], but accurate distribution of sister chromatids over the two daughter cells is essential for the genetic integrity of the organism. Cells thus impose control on the chromosome segregation machinery through a combination of mechanisms known as the spindle checkpoint. Before chromosomes are segregated, the cell must connect each kinetochore to a single spindle pole through a single kinetochore fiber (amphitelic kinetochore attachment; Fig. 1a). This is the only situation that guarantees the fidelity of chromosome segregation, and the cell will attempt to delay anaphase onset if these requirements are not fulfilled. Satisfaction of the mitotic checkpoint marks a point of no return, and overall chromosome movement continues in anaphase even if spindle attachments are disturbed [5,6]; this means that spindle errors can only be corrected within a limited time window, and that undetected kinetochore attachment errors can alter the genetic makeup of daughter cells.

In addition to correct amphitelic attachment, several errors can occur in microtubule/kinetochore coupling (Fig. 1b, c, d). Individual kinetochores might not attach (monotelic attachment), and are left behind once chromosome segregation is initiated at anaphase. Kinetochores of both sister chromatids might attach to microtubules from a single spindle pole (syntelic attachment), and run the risk of segregation into the wrong daughter cell. A single kinetochore might capture microtubules from both spindle poles (merotelic attachment), which places physical stress on the centromere as the microtubules start to pull. The first two errors result in loss of spindle tension, are sensed as a lack of kinetochore stretch, and trigger a strong signal for mitotic checkpoint activation [7]. Merotelic attachments generate kinetochore tension, however, and do not always activate the spindle checkpoint [8-10]. Although merotelic attachments are potentially harmful, they are relatively common in dividing cells, but are normally corrected early in mitosis [11,12]. The control of the mitotic spindle however is deregulated in most carcinomas, resulting in a self-amplifying loop of chromosomal instability. Recent advances underline the importance of spindle defects in the early stages of tumorigenesis, and generate a particular interest in the role of spindle-induced chromosome breakage as the initiator of chromosomal instability [13]. The aim of this paper is to discuss some of the signaling pathways that connect spindle defects, specifically merotelic attachments, to chromosome breakage and the regulation of cell cycle progression.

## Coping with merotelic attachments

Uncorrected merotelic attachments lead to gains and losses of whole chromosomes, termed aneuploidy [11]. In addition, uncorrected merotelic attachments can exert sufficient force to distort individual kinetochores, which damages centromeric chromatin and causes chromosome rupture [13]. The alterations that result from uncorrected merotelic attachments (aneuploidy as well as losses and gains of chromosome arms) are among the most frequently observed genomic defects in cancer [14,15]. Since uncorrected merotelic attachments appear to be common in solid tumors, thery are thought to be a driving force behind the chromosomal instability (CIN) phenotype that accounts for approximately 85% of sporadic carcinomas [16,17]. The chromosome breakage that is associated with uncorrected merotelic attachments generates "reactive" chromosome arms that are able to fuse to intact chromosomes [17]. Such "reactive" arms could initiate the self-propagating chain of instability termed the breakage-fusion-bridge cycle [18]. Whereas the DNA breakage products of uncorrected merotelic attachments, whole chromosome arms, are especially common in low-grade tumors, complex translocation patterns are characteristic of high-grade carcinomas [19,20]. In CIN tumors, uncorrected merotelic attachments might thus initiate genomic instability that is subsequently propagated by breakage-fusion-bridge cycles [17]. Although uncorrected merotelic attachments are common in CIN tumors that show reduced spindle checkpoint control, some healthy cells also bear spindle defects. Genetic techniques using fluorescent probes that flank the centromere showed that a small proportion of normal lymphocytes undergo physical separation of the long and short arms of a single chromosome [21], indicating that some merotelic attachments lead inevitably to chromosome breakage. The uncorrected merotelic attachments responsible for the most important genomic alterations of CIN tumors thus occur occasionally in normal cells.

The prevalence of CIN in cancer and the evidence of uncorrected merotelic attachments in normal cells suggest that correct chromosome segregation is a fundamental problem in evolution, still not fully resolved. Some species, for example *Muntiacus muntjak*, *Potorous tridactylis*, and *Wallabia bicolor *[22-24], assemble their genome in a dozen or fewer chromosomes, with a concomitant reduction in centrosome number. Although low chromosome numbers reduce the number of kinetochores that require control in each cell division, individual kinetochores still form merotelic attachments in *Potorous tridactylis *cells [25]. An extremely low chromosome number nonetheless appears to prevent aneuploidy, thought to be one of the initiating events in tumorigenesis [16,26]. Conditions that readily induce aneuploidy in human and mouse cells only allow for loss or gain of the small sex chromosome Y2 in muntjac cells. Missegregation of the large chromosomes in muntjac is not tolerated due to gene dosage effects [27]. Most mammals must live with the occasional aneuploid cell, however, because they fully depend on spindle dynamics to detect and prevent chromosome missegregation [12,25].

**Figure 2:**
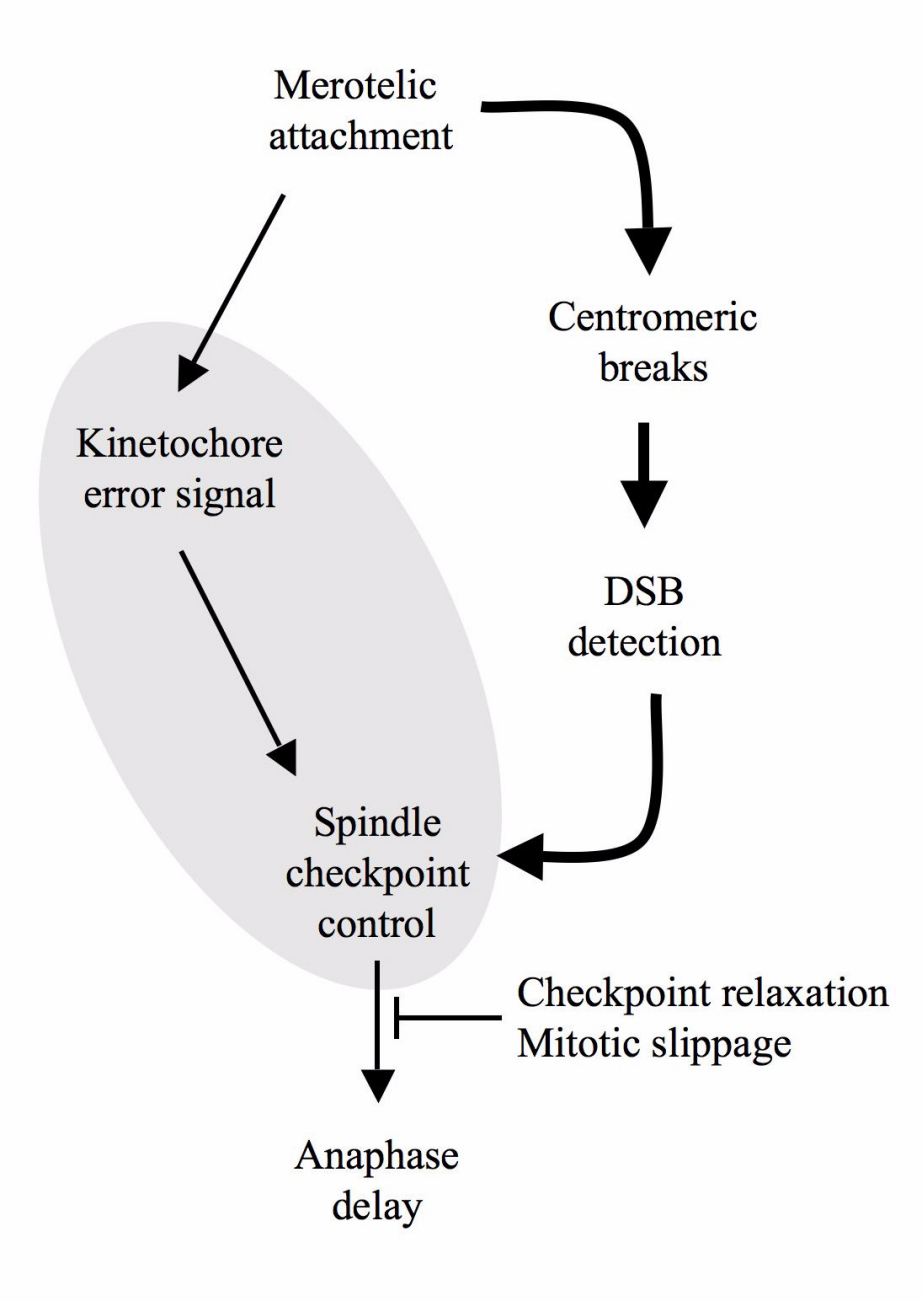
**Signaling by spindle attachment defects**. Two independent pathways act to delay anaphase. The spindle attachment pathway senses kinetochore tension and is especially efficient for detecting monotelic and syntelic attachments, and the DNA damage pathway acts as an additional mechanism that responds to DSB generated by merotelic attachments. When kinetochore attachment defects are undetected, for example in tumors with a CIN phenotype (grey), merotelic attachments and DSB increase, leading to activation of the DNA damage pathway and ultimately to mitotic slippage.

Since the classical mitotic checkpoint fails to detect a proportion of merotelic attachments [8-10], a backup mechanism that detects the consequences of uncorrected merotelic attachments and prevents continuation of mitosis could provide a solution. In addition to aneuploidy, uncorrected merotelic attachments generate chromosome fragments, that is, the formation of double-strand breaks (DSB). DSB could thus indicate a chromosome segregation problem to the cell. Intramitotic DNA damage indeed produces an anaphase delay signal; mammalian cells detect mitotic DNA breaks and respond by activating the spindle checkpoint [28-30]. The crosstalk between break repair and spindle control pathways might have a physiological function in the prevention of aneuploidy, since treatments that induce DNA damage cause aneuploidy in normal cells [31-33]. Although identification of damaged DNA seems a second-best solution, coupling DSB detection to anaphase delay serves the dual purpose of creating a time window for repair and reattaching spindle fibers to the kinetochore (Fig. 2). The situation is more complex in carcinomas that show a weakened response to anaphase delay signals, termed mitotic slippage [16,26]. Mitotic slippage and alterations in the primary detection of kinetochore attachment defects would increase the number of DSB, adding pressure to the detection and repair pathway. Although the break repair pathway might be activated by uncorrected merotelic attachments and the associated DNA damage, it would be ineffective in mitosis if a downstream anaphase delay signal is impaired or bypassed.

## How cells handle chromosome breaks in mitosis

Although merotelic attachments are processed by various pathways, a small proportion escapes detection [21], leaving the daughter cells to deal with a fragmented chromosome. Relaxation of the spindle checkpoint exacerbates this problem [13], placing additional pressure on DNA break repair in CIN tumors. In mammalian cells, double-strand breaks are repaired by two major processes, termed non-homologous end joining and homologous recombination [34]. The availability of repair pathways at the time and subcellular location of intra-mitotic DSB has important consequences; whereas non-homologous end joining repairs breaks by simple religation of two DNA ends, homologous recombination depends on a homologous DNA template. This means that non-homologous end joining can repair DSB throughout the cell cycle, but homologous recombination is virtually inactive in the G1 phase [35]. The DNA breaks caused by uncorrected merotelic attachments are physically the same as other DSB and their centromeric location does not in itself hinder efficient repair [36], but the cell cycle stage in which they are formed obliges the cell to correct DNA damage during or right after mitosis. In addition, some chromosome fragments are sequestered in micronuclei [13], resulting in physical separation from the remainder of chromosomes and precluding homologous recombination.

Mice deficient in any of the DSB repair proteins are generally hypersensitive to induced DNA damage, although they are usually viable [37,38]. Whereas non-homologous end joining or homologous recombination repair mutants have problems repairing induced DSB, the inactivation of a single repair pathway does not result in spontaneous DSB accumulation [13,39,40]. The absence of spontaneous DNA damage in mice lacking a single repair pathway implies that the endogenous DSB formation rate must be relatively low or at least is not life threatening. Notwithstanding the low frequency of spontaneous DSB, many tumors show increased repair system activity, in particular that of non-homologous end joining [41-43]. Non-homologous end joining activation in cancer indicates that DSB are generated at an increased rate, possibly due to chromosome segregation errors and concomitant chromosome arm breakage.

## Non-homologous end joining is essential in a CIN background

Non-homologous end joining appears to be especially important when spindle checkpoint control is relaxed, because the increase in uncorrected merotelic attachments could promote chromosome breakage. In non-homologous end joining, Ku80 is essential for recruitment of repair complexes to DSB, whereas DNA-PKcs is the principal repair kinase [44]. Although residual non-homologous end joining takes place in both Ku80- and DNA-PKcs-deficient cells, *Ku80 *mutation has a far greater impact on DSB repair kinetics than *DNA-PKcs *mutation [45-47]; *DNA-PKcs *disruption thus produces a milder phenotype than *Ku80 *inactivation. Targeted disruption of the *death inducer obliterator *(*Dido*) gene, which causes centrosome amplification and spindle checkpoint relaxation [48], results in a CIN phenotype that includes aneuploidy and chromosome breakage [13]. To determine whether non-homologous end joining is essential in a CIN background, we crossed *Dido *and *Ku80 *heterozygous mice, interbred the double heterozygotes and genotyped all offspring. *Dido *and *DNA-PKcs *heterozygous mice were interbred in the same way. In our crosses, heterozygous and wild-type pups were born at frequencies compatible with normal Mendelian inheritance; we found slightly fewer *Ku80 *and *Dido *mutant newborns (Table 1). In over 1000 pups tested, however, we identified no *Dido Ku80 *double mutants. When double heterozygous *Dido DNA-PKcs *mice were crossed, *Dido *mutants and *Dido DNA-PKcs *double mutants were born at frequencies below the expected ratio, but no marked effect of *DNA-PKcs *mutation was found (Table 2). Although the frequency of *Ku80 *mutants was reduced, some *Dido Ku80 *double mutants would be expected; the absence of these double mutant mice thus indicates synthetic lethality, in accordance with the reported intra-mitotic DSB in the *Dido *mutant [13]. Mutation of *Ku80 *has a far greater impact on DSB repair kinetics than that of *DNA-PKcs *in models of induced DNA damage [49,50], and *DNA-PKcs *also appears to be less important than *Ku80 *in the repair of DSB generated by uncorrected merotelic attachments.

**Table 1:** Combined disruption of *Dido *and *Ku80 *is embryonic lethal.

***Ku80***	***Dido***	N° pups (expected)	N° pups (observed)
+/+	+/+	78.75	147
+/+	+/neo	157.50	278
+/+	neo/neo	78.75	49
+/-	+/+	157.50	216
+/-	+/neo	315.00	411
+/-	neo/neo	157.50	88
-/-	+/+	78.75	24
-/-	+/neo	157.50	47
-/-	neo/neo	78.75	0

Total		1260	1260

Mice heterozygous for *Dido *and *Ku80 *were crossed and all viable pups were genotyped. *Ku80^-/- ^*and *Dido^neo/neo ^*pups were born at a lower frequency than heterozygous littermates. No double mutant mice were born in a population of >1000 animals.

**Table 2:** Combined disruption of *Dido *and *DNA-PKcs*.

***DNA-PKcs***	***Dido***	N° pups (expected)	N° pups (observed)
+/+	+/+	60.25	100
+/+	+/neo	120.50	162
+/+	neo/neo	60.25	22
+/-	+/+	120.50	122
+/-	+/neo	241.00	273
+/-	neo/neo	120.50	40
-/-	+/+	60.25	56
-/-	+/neo	120.50	169
-/-	neo/neo	60.25	20

Total		964	964

Mice heterozygous for *Dido *and *DNA-PKcs *were crossed and all viable pups were genotyped. Whereas the numbers of wild-type, heterozygous and *DNA-PKcs *mutant mice born are as expected, *Dido *and *Dido DNA-PKcs *mutant mouse numbers were below the expected frequency.

**Figure 3:**
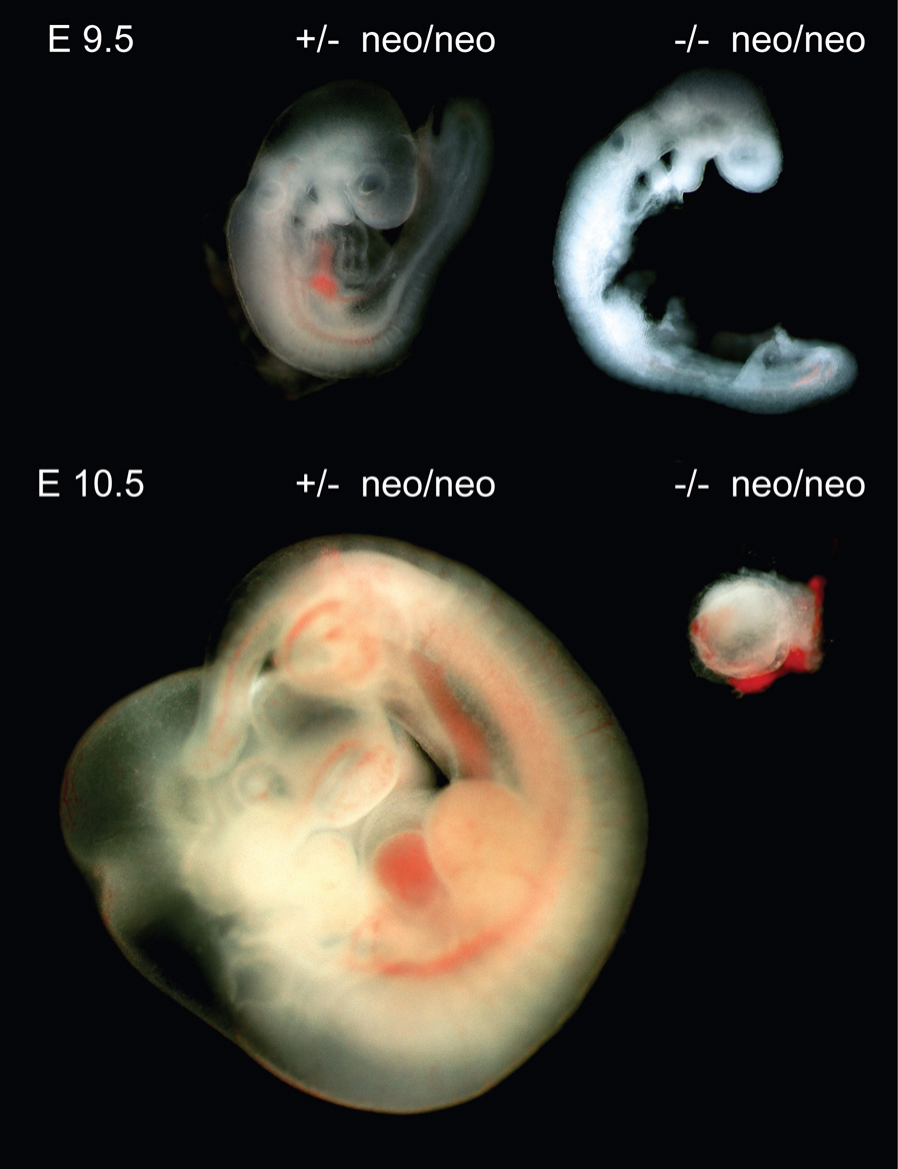
**Combined disruption of *Ku80 *and *Dido *is lethal in mid-gestation**. The figure shows embryos isolated at embryonic day E9.5 (top) and E10.5 (bottom). *Ku80 *heterozygous *Dido *mutant embryos are shown at left and *Ku80 Dido *double mutant embryos at right. At E9.5, double mutant embryos show growth delay in head, heart, and somites. At E10.5, most *Ku80 Dido *double mutant embryos are being resorbed. Magnification, 40-fold. All animal experiments were performed in compliance with EU and CNB animal committee directives.

Since *Dido Ku80 *double mutant embryos die *in utero*, we established the time of gestation at which death occurs. Double heterozygous *Dido Ku80 *mice were interbred and embryos analyzed by dark field microscopy at various times postcoitum. Mutant embryo development was not markedly different from that of heterozygous counterparts up to E8.5 (not shown). Growth delay in *Dido Ku80 *double mutant embryos was first apparent at E9.5, with underdeveloped head, heart and somites (Fig. 3). At E10.5, *Dido *and *Ku80 *single mutant embryos continued to develop normally, whereas most *Dido Ku80 *double mutant embryos had died and were being reabsorbed, and none survived beyond E12.5. Due to variation in survival, we were unable to define an exact time point of death. These data nonetheless show that *Dido Ku80 *double mutant embryos die *in utero *at mid-gestation, suggesting a role for non-homologous end joining in the repair of DNA damage generated by uncorrected merotelic attachments.

## Closing remarks

**Figure 4:**
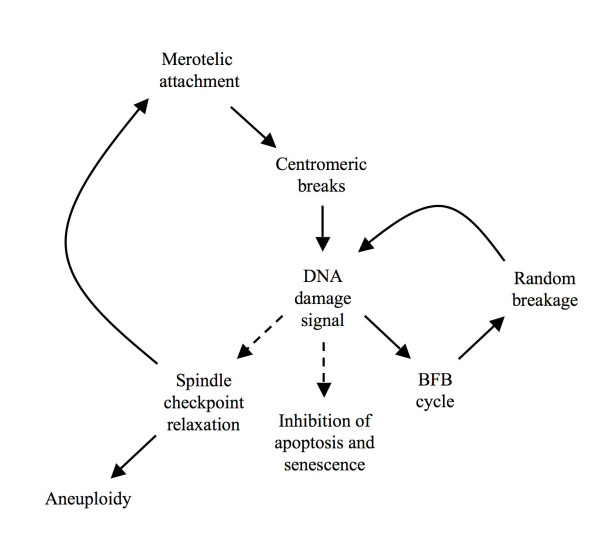
**Model for amplification of chromosomal instability by DNA damage**. Initial merotelic attachments activate DNA damage signaling and inhibit cell proliferation through anaphase delay and induction of apoptosis or senescence. To overcome the block, the downstream spindle checkpoint is suppressed in CIN tumors, increasing the frequency of spindle attachment errors. As a side effect of this continuous breakage, DNA repair mechanisms remain activated, leading ultimately to adaptation through suppression of apoptosis and senescence and through spindle checkpoint relaxation (dashed lines).

Merotelic kinetochore attachments seem to be the Achilles' heel of mammalian cell division, as they can bring about potentially dangerous genomic instability but are poorly recognized by the spindle checkpoint. Even in normal cells, a small proportion of cell divisions thus give rise to chromosome breakage [21]. In the case of intramitotic chromosome breakage, DSB repair systems could transmit a second signal in an attempt to delay mitosis progression [28-30]. The combination of signals involved in the detection of spindle errors has important consequences for cancer development, and gives rise to a working model of early tumorigenesis (Fig. 4).

Any minor alteration in spindle regulation could result in an increase in merotelic attachments that escape detection, giving rise to aneuploidy and chromosome breakage [13]. Breakage activates cellular DNA damage control, shown by increased DSB repair in many tumors [41-43]. The need for non-homologous end joining in a CIN background is emphasized by the synthetic lethality of *Dido Ku80 *double mutants. DNA damage signaling provides feedback to the spindle checkpoint and delays mitosis progression, which prolongs the time window for repair and prevents aneuploidy. Repair by non-homologous end joining not only limits DNA damage and promotes cell survival, but also catalyzes the fusion of reactive chromosome ends. A chromosome fragment generated by spindle defects can thus form end-to-end fusions with normal chromosomes and initiate the breakage-fusion-bridge cycle [18]. Once the breakage-fusion-bridge cycles commence, restoring spindle control no longer ensures stability, since dicentric chromosomes formed by end-to-end fusions can break, even though individual kinetochores are correctly attached [17,18]. A long term effect of DNA damage is cell immortalization; sustained breaks exert selective pressure to evade apoptosis and senescence [51]. Since DSB prevent the progression of mitosis, it is likely that sustained breaks also facilitate mitotic checkpoint relaxation. Continuous mitotic chromosome breakage could thus explain why, over time, CIN tumors become more malignant and refractory to treatment. In conclusion, nature's use of DSB repair systems as a backup for the detection of merotelic attachments might in fact promote chromosomal instability and act as a motor for carcinogenesis. CIN tumors show precisely the characteristics predicted by the above model: Most carcinomas show chromosomal instability and reduced control of the mitotic spindle, combined with enhanced DNA damage repair and reduced apoptotic potential. The challenge for cancer treatment will be to break this vicious circle without causing additional genomic instability.

## Abbreviations

CIN: chromosomal instability; DSB: double strand DNA break.

## Competing interests

The authors declare that they have no competing interests.

## Authors' contributions

AAG performed experiments and analyzed data, CMA designed experiments, KvW wrote the paper. All authors read and approved the manuscript.
